# Orphan Drugs, Compounded Medication and Pharmaceutical Commons

**DOI:** 10.3389/fphar.2021.738458

**Published:** 2021-09-10

**Authors:** Kim Hendrickx, Marc Dooms

**Affiliations:** ^1^ Research Foundation-Flanders (FWO)/Life Sciences and Society Lab, Leuven, Belgium; ^2^ Hospital Pharmacy, University Hospitals Leuven, Leuven, Belgium

**Keywords:** rare diseases, orphan drugs, compounded medication, intellectual property, drug research and development, open science, commons, point of care manufacturing

## Abstract

Regulatory agencies installed orphan drug regulations to stimulate research and development of new innovative treatments for life-threatening diseases with a low prevalence (rare diseases). We established a list of well-known food-related ingredients with clinical evidence for rare diseases in the open medical literature that obtained marketing authorization as an expensive “orphan drug”, protected by intellectual property (IP) rights. We show that these ingredients are part of an established practice of medicinal compounding—a form of point of care manufacturing. We argue that these ingredients should be considered as “pharmaceutical commons”, and that regulatory incentives for private companies and market protection mechanisms such as IP rights are not justified in this case.

## Introduction: Orphan Drugs and Ownership

Worldwide several so-called orphan drug regulations were installed by national agencies to promote research and development (R&D) of medicinal products and devices intended for the prevention, diagnosis and treatment of rare diseases. The European Union defines a drug as “orphan” if it is intended for the diagnosis, prevention or treatment of a life-threatening or chronically and seriously debilitating condition affecting not more than five in 10,000 persons (European Medicines Agency, 2021a). Such a condition is called a “rare disease”. About 30 million people in the EU suffer from a rare disease (Ibid.). Authorization of orphan drugs is subject to European regulation, while pricing and reimbursement is done according to national legislation in the different EU Member States.

This resulted in the authorisation of several hundreds of orphan drugs mainly thanks to the incentives given by national agencies such as market exclusivity, centralized procedure and fee reductions (Huyard, 2009; Mikami, 2017). The main aim of this article is to show that several pharmaceutical ingredients used in orphan drugs are part of an established practice of medicinal compounding and should be considered as “pharmaceutical commons”. We argue that regulatory incentives for private companies and market protection mechanisms such as intellectual property rights are not justified in this case.

There are different ways in which orphan drugs are brought to the market. Over 200 orphan drugs are registered by the European Medicines Agency (EMA) so far, for an estimated total number of between 5,000 and 8,000 rare diseases (European Commission, 2021a). The discovery and development of innovative “new molecular entities” is the least common way. More often, existing drugs are repurposed, which involves lower R&D costs while companies may still benefit from intellectual property (IP) rights. Despite analyses that show the inefficiency today of intellectual property regimes for the drug discovery and development process (Bountra et al., 2017; Lezaun and Montgomery, 2015), ownership remains an issue that pharmaceutical companies cling to, claiming that IP rights are justified in view of the high costs in the drug discovery process (and the high attrition rate), and the ‘small market’ for orphan drugs. More and more analysts now defend the principles of open science, arguing that sharing data early on in the drug discovery process will reduce the duplication of research and investments, and that it will stimulate innovation rather than sticking to a very limited number of ‘promising molecules’ or ‘high impact indications’. Varying business models are proposed, with either limited IP rights or with other forms of ownership (such as non-profit organizations as the owners, see Davies et al., 2017). Extension of the principles of open science to the clinical evaluation of drug candidates and the creation of a system of incentives that would encourage pre-competitive IP-free research was proposed for a radical reconstruction of the drug discovery ecosystem (Bountra et al., 2017; Rubinstein et al., 2020).

Some argue, however, that many practices of sharing data tend to take place amongst a limited number of companies that are motivated by the prospect of obtaining IP rights in the future. In this case, it is not open science but rather a pooling of research data among select companies (excluding other companies or research institutes) in order to make drug discovery more efficient. The final objective is to obtain ownership nonetheless (Lezaun and Montgomery, 2015). When the first orphan drug regulations were established, mainly small and medium sized enterprises created the orphan drug market (e g., Actelion, BioMarin, Genzyme, Shire, SoBi) but today big pharmaceutical industries take the lead (e g., Bayer, Glaxosmithkline, Novartis, Pfizer, Sanofi).

## Compounded Medication

The above analyses and debates are important, but they overlook the practice of pharmaceutical compounding and “hospital exemption” which happens in (hospital) pharmacies, based on pharmacopeial rules, practical know-how and “registries” of clinical evidence. A great many effective treatments for rare diseases exist that are not based on the industrial process of drug discovery and regimes of intellectual property rights (Dooms et al., 2013; Dooms and Carvalho, 2018). Indeed, these compounds did not go first through the EMA for approval but, as they are mentioned in the European Pharmacopoeia^1^, they are allowed by European legislation to be used and administered for helping patients. In some cases, hospital pharmacies also repurpose or reformulate existing drugs and dispense off-label. A number of compounds have been used by companies to obtain an EMA license, thereby turning a relatively cheap and openly available treatment into an expensive commercial product simply by registering it as an orphan drug with the EMA. (Busilvex, Chenodeoxycholic acid, Firdapse, Jorveza, Kolbam, Ledaga, Namuscla, Orphacol, Pedea, Peyona, Siklos, Trisenox, Verkazia, Wilzin). While new drugs need to show a significant benefit over existing ones, no comparisons are made with compounded medication. This problem is well-known and studies exist that compare the costs of compounding with the purchase price of registered orphan drugs (Simoens et al., 2011). When the cheaper compounded alternatives still exist, some countries (like Belgium for example) refuse to reimburse the more expensive product to patients. In other countries (like France and Germany) all medication for rare diseases is fully reimbursed (Picavet et al., 2011; Dooms et al., 2013). In both cases, the situation is not cost-effective, as reimbursement leads to unnecessary costs for public health, and non-reimbursement decreases the sales and profits for commercial products. It is interesting and necessary to look closer into the actual compounds and the practice of compounding, as this provides pathways to think differently about ownership, open science and public-private relationships.

## A List of Common Ingredients: Methods and Results

The data in Table 1 were collected through the Union Register of Orphan Medicinal Products (European Commission, 2021a), WHO Collaborating Centre for Drug Statistics Methodology (World Health Organization, 2021), Medline (2021), The Merck Index (2021), the European Public Assessment Reports (European Medicines Agency, 2021b) and the European Database on Food Additives (European Commission, 2021b).

**TABLE 1:** Ingredients used in the prevention, diagnosis and treatment of diseases with a low prevalence.

Ingredient	Drug Name	Rare Disease	Daily Dose	Preparation	Evidence	Marketing	Food	#Trial P
Mannitol	Bronchitol	Cystic Fibrosis	800 mg	1957	1978	2012	*	642
Betaine	Cystadane	Homocystinuria	6,000 mg	1957	1981	2007	*	—
Cholic acid	Kolbam	Primary Bile acid deficiency	500 mg	1939	1990	2010	*	52
Cholic acid	Orphacol	Primary Bile acid deficiency	500 mg	1939	1990	2010	*	—
Zinc (Sulphate)	Wilzin	Wilson’s Disease	150 mg	1912	1992	2004	—	—
Bromelain	Nexobrid	Burn debridement	Ad Libitum	1961	2010	2012	—	156
L-Carnitine	LevoCarnil	Congenital Urea cycle disorder	2,000 mg	1952	1997	2013	—	—
Folic acid	Folavit	Prevention Spina Bifida	4 mg	1947	1981	1997	*	—
Biotin	Biotine	Acylcoen A dehydrogen deficiency	—	1976	—	NA	*	—
Cannabidiol	Epidyolex	Lennox Gastaux Syndrome	—	1964	2013	2019	—	715
Riboflavin	Riboflavine	Acylcoen A dehydrogen deficiency	—	1935	2003	NA	*	—
pThyroid Horm	Natpar	Hypoparathyroidism	0.1 mg	—	1950	2017		124
L-Citrulline	N A	CPS and OCT-deficiency	100 mg/kg	1938	—	NA	—	—
Creatine	N A	GAMT-deficiency	—	1868	2002	NA	—	—
L-Cysteine	N A	—	—	1926	—	NA	*	—
D-Mannose	N A	Congenital Deficiency Glycosilation	600 mg/kg	1941	1997	NA	—	—
D-Ribose	N A	AMP-deaminase deficiency	—	1909	1991	NA	—	—
Glycine	N A	Diagnostic	100 mg/kg	1924	—	NA	*	
Uridine	N A	Pyrimidine pathway deficiency	—	1931	—	NA	—	—
Beta-carotene	N A	Erythropoethische Protoporfyria	300 mg	1950	1980	NA	*	—
Coenzyme Q 10	N A	Mitochondrial defects	—	1979	—	NA	—	—
L-Arginine	N A	Congenital Urea cycle disorder	—	1943	1969	NA	—	—
Magnesium salts	NA	Gitelman syndrome	—	NA	2008	NA	—	—

Table 1 shows a list of ingredients that are traditionally not considered as medicine but used as food additives or supplements (such as amino-acids and vitamins) or hormones, and which became expensive branded products through a registry procedure with the EMA. Repurposed or repositioned drugs are also used in the treatment of rare diseases (drug repurposing is the discovery of a new use for already approved or investigational medicinal products). We have left these repositioned ingredients out of this table as they were initially intended for medical use after extensive medical research and only got a second life in rare diseases (Tambuyzer et al., 2020; Picavet et al., 2011). We have focused on traditionally ‘non-medical’ ingredients (“ingredient” in Table 1) used in the prevention, diagnosis and treatment of rare diseases that were discovered many years ago (“preparation” in Table 1), gained proven clinical benefit in the open medical literature (“evidence” in Table 1) and obtained a marketing authorization by EMA (“Marketing” in Table 1) on the basis of historical data and a limited number of clinical trials (“#Trial P” in Table 1). Several of these ingredients are allowed today as food additives or supplements (“Food” in Table 1). Ingredients without a Drug Name (NA in Table 1) are compounded by pharmacists and are not marketed (yet) in the EU.

## Discussion: Commons in Relation to Open Science

As indicated in the first section, there are many debates going on about IP rights and alternative innovation and/or ownership models. Pleas are made to apply the principles of open science (in analogy to open software) in drug research and development. We support such pleas but we also wish to draw attention to a practice that runs parallel to the processes of industrial drug discovery and development. This practice is that of pharmaceutical compounding, where pharmacists make custom medicinal formulations to fit the needs of their patients, based on shared clinical experience, often using substances that are, strictly speaking, not even “drugs” but food additives or supplements. One angle to look at this practice is to consider it a special case of “open science”. Yet, open science is mostly used to discuss industrial drug development and innovation pathways. The case of pharmaceutical compounding with common food ingredients (additives or supplements) is not the same kind of innovation, nor the same kind of science. The relation between a *community* of professionals using *common* ingredients can be more adequately described with the historical and political notion of “the commons”. In modern times the commons were the common and natural resources accessible to all members of a society, held in common but not owned privately and managed for individual or collective benefit. Collective systems of managing common land in late medieval England is a well-known example. In the 16–18th Centuries, policies of “enclosure” gradually placed these collectively owned and exploited lands in private hands. These policies were later applied at a global scale. Posthoc rationalizations of these policies are based on the idea that the commons are untenable, because abuse and overexploitation will occur at some point (Hardin, 1968). This thesis has been disproven, and many examples of successful ‘commons’ have been analyzed by Nobel-prize winning economist Elinor Ostrom (1990). Today, there is a resurgence of the commons in areas ranging from open software to common urban spaces and the reclaiming of rights by indigenous peoples (Gutwirth, 2018). We argue in this paper that an important number of pharmaceutical ingredients should be conceived of and regulated as commons.

All of the ingredients in Table 1 were discovered many years ago (between 1868 and 1976) and gained proven clinical evidence (through experience and serendipity) for a rare disease in the open medical literature (between 1978 and 2013) before their marketing by a private sponsor as an “orphan drug”. These ingredients can be considered as res publicae or commons without any reason for market protection on the basis of historical data and a limited number of clinical trials. All the ingredients in Table 1 obtained clinical evidence in the open medical literature but got market access with incentives that did not encourage basic innovative research but rather (ab)used it.

The advantage of referring to the notion of the commons is a change in perspective—another way of approaching a particular existing practice that is driven by a different rationale than that of ‘sharing’ for the sake of innovation. An approach in terms of commons and “commoning” (as an activity) provides a better sociological description of practices of pharmaceutical compounding. A sociological description is important, because it allows emphasizing specificities of compounding that remain unnoticed and unexploited in technical debates about drug discovery, development and systems of innovation.

A first specificity to be observed is the compound itself. In the case of food ingredients, pharmacists “repurpose” a substance that is not a drug. However, in certain patients, these ingredients have clinical effects. As with food allergies, where “harmless” ingredients provoke reactions that may be life-threatening, this shows that the boundary between food and drugs is often less clear in the clinic than in the realm of law (Hendrickx, 2019). While that legal boundary exists for good reasons (such as safety), legislation also leads to absurd situations where a common ingredient may change status and become more expensive because it is recognized as a drug for a specific rare disease. Food additives and supplements are the object of separate legislation for safety aspects but this legislation is unrelated to pharmaceuticals and medicine.

A second specificity is the community in which compounding takes place. Debates on open science and innovation tend to focus on R&D structures such as public-private partnerships and specific business models. We hereby overlook the know-how and practices that take place amongst pharmacists and doctors in clinical settings: the sharing of “recipes”, evidence and experience. While hospitals are also profit-seeking structures and subject to budgetary rationalization under national health and economic policies, the medical personnel and pharmacists have a primary interest in helping or curing patients. For rare diseases, medication tailored to a patient’s unique needs is often necessary and medical compounding is a form of what is called “point of care manufacturing” (though the term is today often reserved for the medical use of 3D printing). The hospital pharmacists gain no financial benefit from the practice of pharmaceutical compounding. The commitment to sharing recipes and keeping evidence-based records of treatments is not an innovation strategy, but a logical part of a common effort to help patients with rare disorders. In her famous analysis of case studies of contemporary practices in managing common lands and resources, economist Elinor Ostrom (1990) shows that a shared responsibility fuels the commitment of individuals and groups in managing their specific commons. She disproved the thesis that collective management sooner or later leads to abuse and overexploitation. If we consider the fact that pharmaceutical compounding is a practice of shared responsibility to make good use of commonly available ingredients, then this is indeed a practice of self-governing a “pharmaceutical commons”, as termed by Lezaun and Montgomery (2015) in a different context. This poses the question whether it would not be better to invest time and resources in establishing common databases (Dooms and Carvalho, 2018), methods, and recipes instead of evaluating private reformulations of these very same and common substances by the personnel of the EMA. Though not always reimbursed by public health authorities and social security, it is today perfectly legal to file applications for molecules that are part of a pharmaceutical commons, resulting in what can be called the “enclosure” of common therapies. If this were to be prohibited, then private companies would have to go and invest where their infrastructure and know-how are more useful—the development of new innovative molecular entity drugs—and this is a domain where the challenges of open science, innovation and alternative business models and partnerships merit our full attention indeed.

## Conclusion

Regulatory agencies installed orphan drug regulations to stimulate research and development of new innovative treatments for life-threatening diseases with a low prevalence (rare diseases). We established a list of well-known food-related ingredients with clinical evidence for rare diseases in the open medical literature that obtained marketing authorization as an expensive “orphan drug”, protected by intellectual property (IP) rights. We have argued that these ingredients are part of an established practice of medicinal compounding and should be considered as “pharmaceutical commons”, and that regulatory incentives for private companies and market protection mechanisms such as IP rights are not justified in this case. In addition, the concept of the commons brings to light the practice of medicinal compounding—a form of point of care manufacturing - which is not profit-driven, but done by a skilled community of pharmacists with proper resources and evidence-bases who seek to cure patients with rare diseases. This is often overlooked in debates about open science and innovation and it provides a welcome opportunity to shift technocratic debates about IP, regulatory incentives and innovation models to the knowledge, expertise and resources that exist within pharmaceutical communities in the face of rare diseases.

## Footnotes

1
https://www.edqm.eu/en/european-pharmacopoeia-ph-eur-10th-edition

## Abbreviations

EMA, European Medicines Agency; EU, European Union; IP, Intellectual property; NA, not applicable; R&D, Research and Development; WHO, World Health Organization.

## References

[B1] Bountra C. Lee W. H. Lezaun J. (2017). A New Pharmaceutical Commons: Transforming Drug Discovery. Oxford: Oxford Martin Policy Paper, 1–28.

[B2] Davies E. H. Fulton E. Brook D. Hughes D. A. (2017). Affordable Orphan Drugs: a Role for Not-for-profit Organizations. Br. J. Clin. Pharmacol. 83, 1595–1601. 10.1111/bcp.13240 28109021 PMC5465340

[B5] Dooms M. Carvalho M. (2018). Compounded Medication for Patients with Rare Diseases. Orphanet J. Rare Dis. 13, 1. 10.1186/s13023-017-0741-y 29301541 PMC5753439

[B6] Dooms M. Pincé H. Simoens S. (2013). Do we Need Authorized Orphan Drugs when Compounded Medications Are Available?. J. Clin. Pharm. Ther. 38, 1–2. 10.1111/jcpt.12006 22973866

[B7] European Commission (2021b). Food Additives. Available at: https://webgate.ec.europa.eu/foods_system/main/?sector=FAD&auth=SANCAS (Accessed July, 2021)

[B8] European Commission (2021a). Public Health - Union Register of Medicinal Products. Available at: https://ec.europa.eu/health/documents/community-register/html/index_en.htm (Accessed July, 2021)

[B9] European Medicines Agency (2021b). European Public Assessment Report. Available at: https://www.ema.europa.eu/en/glossary/european-public-assessment-report (Accessed July, 2021)

[B10] European Medicines Agency (2021a). Orphan Designation: Overview. Available at: https://www.ema.europa.eu/en/human-regulatory/overview/orphan-designation-overview (Accessed August, 2021)

[B11] Gutwirth S. (2018). Quel(s) Droit(s) Pour Quel(s) Commun(s) ?. Revue Interdisciplinaire d’Etudes Jurtdiques 81, 83–107. 10.3917/riej.081.0083

[B12] Hendrickx K. (2019). The Political Space between Words and Things: Health Claims as Referential Displacement. Sci. as Cult. 28 (4), 427–448. 10.1080/09505431.2018.1557629

[B13] Huyard C. (2009). How Did Uncommon Disorders Become 'rare Diseases'? History of a Boundary Object. Sociol. Health Illn 31 (4), 463–477. 10.1111/j.1467-9566.2008.01143.x 19397760

[B14] Lezaun J. Montgomery C. M. (2015). The Pharmaceutical Commons: Sharing and Exclusion in Global Health Drug Development. Sci. Technol. Hum. Values 40 (1), 3–29. 10.1177/0162243914542349 PMC4361701 25866425

[B15] Mikami K. (2017). Orphans in the Market: the History of Orphan Drug Policy. Soc. Hist. Med. 32 (3), 609–630. 10.1093/shm/hkx098 31384102 PMC6664588

[B18] Ostrom E. (1990). Governing the Commons. The Evolution of Institutions for Collective Action. Cambridge: Cambridge University Press.

[B19] Picavet E. Dooms M. Cassiman D. Simoens S. (2011). Drugs for Rare Diseases: Influence of Orphan Designation Status on price. Appl. Health Econ. Health Pol. 9 (4), 275–279. 10.2165/11590170-000000000-00000 21682354

[B20] Rubinstein Y. R. Robinson P. N. Gahl W. A. Avillach P. Baynam G. Cederroth H. (2020). The Case for Open Science: Rare Diseases. JAMIA Open 3, 472–486. 10.1093/jamiaopen/ooaa030 33426479 PMC7660964

[B21] Simoens S. Cassiman D. Picavet E. Dooms M. (2011). Are Some Orphan Drugs for Rare Diseases Too Expensive? A Study of purchase versus Compounding Costs. Drugs Ther. Perspect. 27, 24–26. 10.2165/11601640-000000000-00000

[B22] Tambuyzer E. Vandendriessche B. Austin C. P. Brooks P. J. Larsson K. Miller Needleman K. I. (2020). Therapies for Rare Diseases: Therapeutic Modalities, Progress and Challenges Ahead. Nat. Rev. Drug Discov. 19 (2), 93–111. 10.1038/s41573-019-0049-9 31836861

[B23] The Merck Index (2021). The Merck Index* Online. Available at: https://www.rsc.org/merck-index.

[B24] World Health Organization (2021). ATC/DDD Index. Available at: https://www.whocc.no/atc_ddd_index/(Accessed July, 2021).

